# The effectiveness of REBT in reducing occupational stress among agricultural education lecturers: Implications for school management team

**DOI:** 10.1097/MD.0000000000048652

**Published:** 2026-05-15

**Authors:** Benedicta Anene Omeje, Ngozi Sandra Ikenye, Felicia Ngozi Ezeaku, Emeka Kelechi Ogbonna, Kelechi Ruth Ede, Ifunanya Nkechi Ohamobi, Gloria Njideka Ikegbusi, Scholastica Uchenna Ekwueme, Godwin E. Eze, Mary Oluwatoyn Ani, Bassey Ndubisi Njioku, Ebele G. Nwoye, Fredrick C. Onah, Lilian U. Ekenta, Njideka Chinwe Ugoji, Samuel Ifeanyi Aba, Robert Nyakuwa

**Affiliations:** a Department of Agricultural Education, Faculty of Vocational and Technical Education, University of Nigeria, Nsukka, Enugu State, Nigeria; b Faculty of Education, Delta State University, Abraka, Delta State, Nigeria; c Department of Educational Foundations, University of Nigeria, Nsukka, Enugu State, Nigeria; d Department of Educational Foundations, Faculty of Education, Chukwuemeka Odumegwu Ojukwu University, Igbariam, Anambra State, Nigeria; e Harare Institute of Technology, University of Stellenbosch, Stellenbosch, Zimbabwe.

**Keywords:** educators, rational emotive behavior therapy, REBT, universities, work stress

## Abstract

**Background:**

Lecturers have a multitude of responsibilities to fulfill, and how they perceive their workload can greatly impact their performance and well-being. One factor that can influence teaching quality and overall satisfaction is the perception of excessive workload. Keeping this in mind, this study aimed to explore the impact of rational emotive behavior therapy on work stress and perceptions among Lecturers.

**Methods:**

The study was conducted in public universities in Southeast Nigeria using an experimental design with a randomized trial control approach. A total of 46 lecturers in agricultural education at public institutions. The primary measure used was a stress scale. Participants underwent a 16-session therapy program over a period of 2 months, introducing them to rational emotive behavior therapy (REBT) through an intervention manual. Data collected during the pretest and posttest phases were analyzed using Analysis of Covariance and descriptive statistics (mean, chi-square, and percentage).

**Results:**

The results indicated that lecturers in the REBT group experienced a significant decrease in occupational stress compared to the control group both during and after treatment. This suggests that REBT was more effective in reducing work stress among agriculture education lecturers than the control group. Additionally, male lecturers showed a greater reduction in stress levels during treatment than their female counterparts. Age was not found to have a significant impact on stress levels among agriculture education professors.

**Conclusion:**

The study suggests that REBT is an effective therapeutic approach for managing work-related stress among instructors in agricultural education at universities in Southeast Nigeria. Based on these findings, the study recommends that REBT practitioners consider using this modality to assist college and university teaching staff in stress management.

## 1. Introduction

Agriculture is the foundation of any developing economy, providing essential resources for humanity and raw materials for manufacturing. According to the World Bank Group,^[1]^ agriculture is crucial for ending extreme poverty, promoting shared prosperity, and feeding an expected 9.7 billion people by 2050. Agricultural education imparts various skills, such as crop production, animal care, soil and water conservation, and marketing.

Agricultural education instructors have diverse responsibilities, some of which can be challenging and may conflict with their primary role as educators. These responsibilities include organizing seminars, tutoring sessions, and laboratory classes, on top of preparing and delivering lectures to both graduate and undergraduate students. Professors in agricultural education also engage in practical tasks related to their field, such as assisting students in raising animals and cultivating crops. The additional duties, like the sandwich program and postgraduate courses, can be stressful, leading to health issues.^[2]^

Occupational stress has been associated with reduced work performance, absenteeism, anxiety, and an increased risk of accidents in and out of the workplace.^[3]^ Additionally, workplace maladjustment and occupational health problems may arise from occupational stress.^[4]^

Workplace stress is a common issue for people worldwide.^[5-9]^ These issues affect workers’ physical and mental well-being and may lead to a decline in productivity and job satisfaction as well as a rise in turnover.^[8]^ Lecturers in academic contexts frequently experience high levels of work stress due to the demands of both teaching and research. This is especially noticeable at Nigerian colleges, where instructors struggle with factors such as low pay, overwork, and a lack of funding.^[8]^ According to Aina and Adeleke,^[9]^ university instructors in Nigeria face high levels of work-related stress due to reasons such as low pay, a rigorous workload, and a lack of support.

Perceptions of workload and coping mechanisms often differ by gender. According to McKeand,^[10]^ gender may impact how people respond to stress. Women are more prone to emotional outbursts because stressful conditions activate the emotional processing centers of the female brain. Onu et al^[11]^ found that women in agricultural education report more weariness than their male peers. It is unknown how and to what extent coping mechanisms are employed at this research site.

While some instructors may view difficult situations negatively, others are able to understand them more accurately. Irrational beliefs are negative reactions that people cling to during stressful times, and they are the main cause of human dysfunction.^[12]^ By challenging these beliefs, individuals can shift from negative to positive emotional responses. Feelings of worry, depression, and emotional distress can stem from irrational thinking.^[12,13]^ Therefore, occupational stress can affect work-related behaviors, home life, health, and productivity. Thus, active psychotherapies such as rational emotive behavior therapy (REBT) are required.

To help people live better lives by addressing emotional and behavioral issues, Albert Ellis developed rational emotive behavior therapy (REBT), a subset of cognitive behavioral therapy (CBT). A key component of REBT, according to Ellis’ model, is that people are emotionally disturbed by how they construct their perceptions of unfavorable circumstances through language, evaluative beliefs, meanings, and philosophies about the world, themselves, and others, rather than the unfavorable circumstances themselves.^[12]^ According to the author, REBT is based on the notion that many people who have emotional or behavioral issues do so because of how they interpret their experiences rather than merely the actual occurrences.

After that, REBT is employed as a teaching method in which the therapist regularly gives the client direct instruction on how to identify irrational and harmful beliefs and attitudes.^[14]^ Rigid, unrealistic, illogical, and absolutist perceptions of stressful situations could be changed with more self-helping and logical ones.

ABCDE is used to further illustrate REBT assumptions. Kurasaki and Terjesen^[15]^ state that the ABCDE model, which illustrates the explanatory process of emotional disturbance, is the main focus of REBT. The ABCDE model assists the person in seeing that their perception and assessment of some of these unpleasant life situations, rather than the events themselves, is what causes the negative affective reaction (such as stress, despair, or rage). The ABCDE paradigm of REBT is summed up as follows by Dryden and Branch^[14]^: activating event/adversity is represented by A, irrational belief by B, emotional and behavioral consequences by C, disputes or arguments by D, and new effect by E. After effectively disproving the false notion, further effects will become apparent. The ABCDE model can be very useful in tracking the emergence of a false belief and offering a high-level overview of how to refute and replace it.^[14]^ Applying this model to agricultural education teachers could be helpful and useful in refuting illogical views they may have formed that are related to occupational stress.

### 1.1. Gaps

Although REBT has been extensively studied for mental health and stress management in other parts of the world, the effect of REBT on occupational stress among Nigerian university professors, particularly in agriculture education, has not been well studied empirically. Few studies have specifically addressed the lecturer populations in Nigeria when it comes to the literature on the mental health of agricultural education teachers. Further research is needed to determine whether REBT can successfully manage stress in the particular setting of Nigerian higher education. To assess the efficacy of this intervention in this particular demographic, which currently lacks specialized attention, it is imperative to close this gap. Thus, the researchers investigated the effect of rational emotive behavior therapy on occupational stress among agricultural educators in universities in Southeast Nigeria.

## 2. Materials and methods

### 2.1. Ethical consideration

The approval and every protocol to conduct this research work were obtained from the Strategic Contacts, Ethics, and Publication (STRACEP) Committee of the University of Nigeria with UNN/EC/STA/010/FE=RC101. We equally conducted this research in line with the American Psychological Association’s established ethical principles and standards. This trial (study) was retrospectively registered in the UMIN Clinical Trial Registry with registration number: UMIN000045204.

### 2.2. Design

This study is a randomized controlled trial, conducted in southeast Nigeria.

### 2.3. Participants and procedure

A total of 46 agricultural university lecturers participated in the study. These were eligible participants identified using the stress and illogical beliefs measures. Other eligibility criteria included:

- Being employed as a lecturer in agricultural education,
- Being a lecturer for not <5 years,
- With a minimum qualification of a bachelor’s degree in an educational course,
- Signing the consent letter, and
- Not having received psychological or counseling orientations within a space of 7 years.

**Figure 1.**
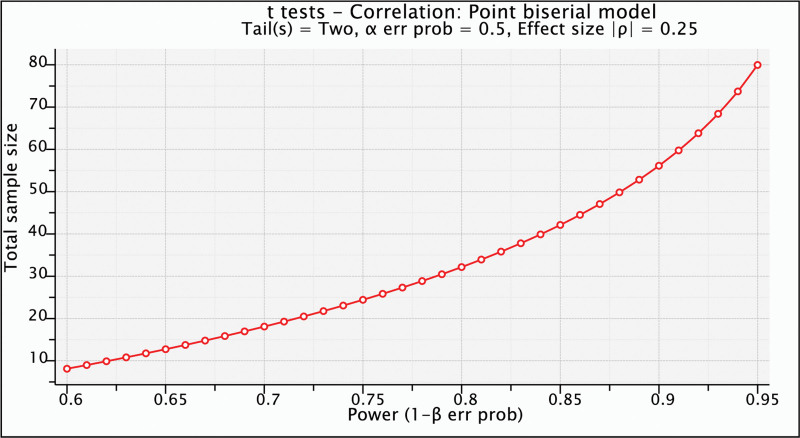
Sample calculation.

Those who did not meet the stated criteria were excluded from the study. Of all the 46 eligible participants, 6 lecturers were from ABUU, 7 from EBUA, 9 ESUST lecturers, 13 MOUAU lecturers, and 11 UNN lecturers, with an age range of 18 to 70 years. A total of 22 lecturers were males (treatment = 12; control = 10), while 24 people were females (treatment = 13; control = 11) are contained in Table 1. The sample power (effect size = 0.25, α = 0.5, power = 0.85) was determined using GPower 3.1 software (see Fig. 1).

**Table 1:** Pretest/posttest of the effect of REBT and counselling service on occupational stress among agricultural education lecturers.

		Pretest			Posttest		
Groups	N	$\bar{x}$	SD	$\bar{x}$		SD	Mean difference
REBT	25	66.56	1.80	42.72		1.59	23.84
Counselling service	21	66.23	2.52	53.23		3.33	13.00

REBT = rational emotive behavior therapy, SD = standard deviation.

**Figure 2.**
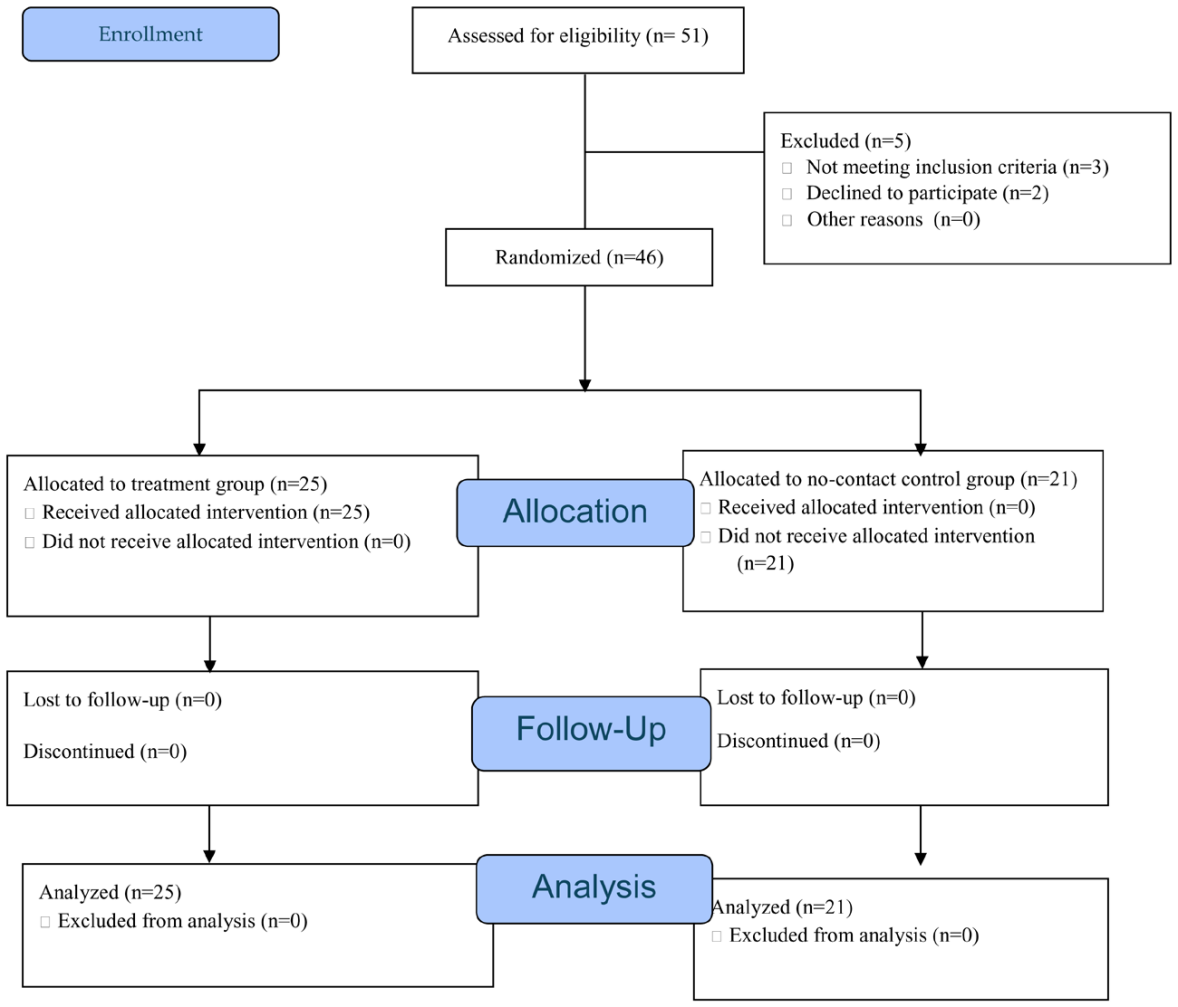
Participants’ consort diagram.

The research team produced a set of even numbers and odd numbers. Participants that picked an even number were assigned to the treatment group, while participants that picked an odd number were assigned to the control group. What the odd and even numbers were not made known to the participants, ensuring that they did not determine group they belonged to. In fact, it was to ensure that concealment was maintained. A total of 25 participants were assigned to the treatment group, while 21 were in the control group. Intervention was given to the participants in the treatment group. Figure 2 demonstrated the participants’ group assignment.

After the intervention package, a posttest (time 2) was administered to all participants in the intervention and the waitlist control group. At each assessment, the researchers and research assistants (counsellors) were distributed and retrieved on the spot from the participants during each assessment. To achieve optimum compliance among the participants, the researcher subsidized the hotel accommodation of the participants up to 50% as well as the light refreshment at each meeting.

### 2.4. Intervention

Rational emotive behavioral therapy is a psychological intervention programme targeted at restructuring the cognitive errors and challenging emotional dysfunctions responsible for occupational stress among university agricultural lecturers. Fundamentally, the intervention approach was designed to help the university agricultural lecturers achieve basic rational emotive skills and techniques for effective management of occupational stress and challenge the irrational beliefs associated with teaching/occupational stress. During the development of this study manual, previous literature related to occupational stress was revealed.^[16]^ Copies of the treatment manual were carefully verified by REBT experts and counselor. The treatment package was developed to run through 8 weeks of 16 sessions with 1 hour per session that is, twice per week. Several occupational stress techniques were employed during the intervention. like, desensitization, cognitive restructuring, considering alternative explanations (validity testing), thought-stopping techniques, disputing irrational beliefs, and forceful self-statements and procedures adopted by previous studies.^[16,17]^

### 2.5. Conventional control counselling

By the time members of the control group started their therapy, those in the treatment group had already begun theirs. One of the University counselors was assigned to implement the sessions designated for the participants in the control group. The participants in the control group were exposed to a different treatment package that aimed at work-family conflict. This helped the researchers understand the initial group difference with regard to treatment effectiveness. They received 3 points (time 1 and time 2) same time and day.

### 2.6. Measures

#### 2.6.1. *Teachers Occupational Stress Questionnaire (TOSQ*)

Teachers occupational stress was developed by Hendreş et al.^[18]^ TOSQ is a 20-item instrument that measures the rate of occupation stress among teachers. The items were rated on a 6-point Likert scale, with responses ranging from 1 – *this activity does not stress me at all* to 6 – *this activity stresses me very much*. Several studies have validated the effectiveness of TOSQ in measuring occupational stress among teachers’ population.^[19]^ In this study, the internal consistency of the instrument shows 0.90, indicating the validity of the instrument.

## 3. Results

The result in Table 1 shows the pretest and posttest mean scores of the effects of rational emotive behavior therapy (REBT) and counseling service on occupational stress among agricultural education lecturers in Southeast Nigerian universities. The result showed that the group exposed to REBT had a pretest occupational stress mean score of ($\bar{x}$ = 66.56, SD = 1.80) and a posttest mean score of ($\bar{x}$ = 42.72, SD = 1.59). The mean difference was 23.84. The result also shows that the group exposed to placebo services had a pretest mean score of ($\bar{x}$ = 66.23, SD = 2.52) and a posttest mean score of ($\bar{x}$ = 53.23, SD = 3.33). The mean difference was 13.00. The mean difference of 23.84 and 13.00 for those exposed to REBT and Conventional Control Counselling respectively shows that REBT was more effective in reducing the occupational stress of agricultural education lecturers than the comparison group.

Table 2 displays the ANCOVA of the impact of conventional control counseling (control group) and rational emotive behavior therapy on occupational stress among agriculture education professors at universities in Southeast Nigeria. An *F*-ratio of (*F* (1, 46) = 227.202, *P* < .05, $\eta_{p}^{2}$ = 0.84) was achieved, according to the results. The null hypothesis was rejected because the associated probability value of .00 is less than the significance level of .05. Therefore, it can be concluded that agricultural education teachers at institutions in Southeast Nigeria had a notable reduction in occupational stress as a result of rational emotive behavior therapy. Compared to the counseling service, REBT considerably lowers lecturers’ occupational stress. The result further showed the effect size of ($\eta_{p}^{2}$ = 0.84), which indicates that 84 percent (84%) variance of the reduction in occupational stress level among agricultural education lecturers was due to the therapy.

**Table 2:** Analysis of covariance (ANCOVA) of the effect of REBT on occupational stress among agricultural education lecturers in Southeast Nigerian universities.

Source	Type III sum of squares	Df	Mean square	*F*	Sig.	Partial eta squared
Corrected model	1323.980	4	330.995	61.268	0.00	0.85
Intercept	120.479	1	120.479	22.301	0.00	0.35
Pretest TOSQ	0.632	1	0.632	0.117	0.73	0.00
Groups	1227.434	1	1227.434	227.202	0.00	0.84
Gender	17.822	1	17.822	3.299	0.07	0.07
Groups gender	44.721	1	44.721	8.278	0.01	0.16
Error	221.498	41	5.402			
Total	1,05,428.000	46				
Corrected total	1545.478	45				

$\eta_{p}^{2}$ = partial eta squared.

ANCOVA = analysis of covariance, REBT = rational emotive behavior therapy, TOSQ = Teachers Occupational Stress Questionnaire.

Results in Table 3 show the pretest and posttest mean scores of the influence of gender on occupational stress among agricultural education lecturers in Southeast Nigerian universities. Results showed that the male lecturers had a pretest occupational stress mean score of ($\bar{x}$ = 66.77, SD = 2.04) and a posttest mean score of ($\bar{x}$ = 46.90, SD = 4.84). The mean difference was 19.87. The result also shows that the female lecturers had a pretest mean score of ($\bar{x}$ = 66.08, SD = 2.22) and a posttest mean score of ($\bar{x}$ = 48.08, SD = 6.71). The mean difference was 18.00. The mean difference of 19.87 and 18.00 for male and female lecturers respectively shows that the occupational stress level of male lecturers reduced during the treatment period more than their female counterparts. To test whether gender significantly influences occupational stress among agricultural education lecturers in Southeast Nigerian universities.

**Table 3:** Influence of gender on occupational stress among agricultural education lecturers.

		Pretest			Posttest		
Gender	N	$\bar{x}$	SD	$\bar{x}$		SD	Mean difference
Male	22	66.77	2.04	46.90		4.84	19.87
Female	24	66.08	2.22	48.08		6.71	18.00

SD = standard deviation.

Table 2 displays the ANCOVA of the impact of gender on occupational stress among teachers in agriculture education at universities in Southeast Nigeria. The findings indicate that an *F*-ratio of 3.299, *P* > .05, and $\eta_{p}^{2}$ = 0.07 was attained. Considering that the associated probability value of 0.07 is higher than the significance level of 0.05, the null hypothesis was not rejected. Therefore, it may be concluded that there was no discernible impact of gender on occupational stress among teachers of agricultural education in universities in Southeast Nigeria. Only 7% of the variance in the decrease in occupational stress levels among agricultural education lecturers was attributable to gender, according to the results, which also revealed an impact size of $\eta_{p}^{2}$ = 0.07.

## 4. Discussion

Rational emotive behavior therapy (REBT) was found to be more successful than traditional control therapy in lowering the occupational stress of agriculture education lecturers. In Southeast Nigerian universities, agriculture education instructors’ occupational stress levels were significantly impacted by REBT. Additionally, compared to their female counterparts, male lecturers’ occupational stress levels decreased more during the treatment period, according to the study. The effectiveness of REBT was validated by the fact that among agricultural education instructors in universities in Southeast Nigeria, gender had no discernible impact on occupational stress. Results showed that, when compared to the waitlisted control group, agricultural professors who got psychological treatment had a considerably lower cause–effect connection of occupational stress as a result of REBT. At follow-up, the effect of REBT persisted, and instructors at agricultural universities credited REBT with lowering their occupational stress levels. The results of the study provide empirical evidence that REBT can transform participants’ negative ideas and thoughts into more reasonable ones. Prior research has also confirmed that REBT is a useful tool for lowering illogical thinking. For instance, REBT significantly improved the work capacity and occupational stress of electronics workshop instructors, according to Ogbuanya et al.^[4,20]^ When compared to members of a control group, Eze^[21]^ found that REBT was effective in reducing the work-related stress of special education teachers. Similarly, it was shown that a rational-emotive health education intervention was helpful in helping technical college professors manage their stress and illogical views.^[22]^

Cognitive behavioral therapy (CBT) significantly reduced occupational stress among science and social science education facilitators in a related study by Ugwuanyi et al.^[23]^ Additionally, REBT was shown to be beneficial in helping special education instructors in Nigerian elementary schools manage their stress and irrational beliefs.^[24]^ Participants in an additional study by Onyishi et al^[25]^ on the use of rational emotional occupational health coaching in the management of police subjective well-being and job ability demonstrated a notable improvement. Research has demonstrated that systematic cognitive behavioral therapy (CBT) lowers depression, OCD, and anxiety.^[26]^ Dalgaard et al^[27]^ reported that CBT significantly improved patients’ long-term return to work when they had stress-related problems.

After being exposed to Rational Emotive Cognitive Behavioral Coaching, participants with high depressive mood showed a considerable improvement, according to Eseadi et al.^[28]^ According to research, cognitive behavioral therapy (CBT) is the best way to cure irrational thinking.^[29]^ The study’s conclusions showed that, in comparison to the waitlist group exposed to traditional lecture techniques, agricultural lecturers exposed to REBT had a higher mean rating. The findings demonstrated that REBT considerably increased academic staff members’ work-related stress. A well-packaged REBT could assist people in reframing irrational ideas that might influence their future objectives and in forming new, reasonable beliefs.^[30]^ According to the reviewed literature, REBT is a practical and all-encompassing teacher intervention that can support self-realization and emotional well-being on both a social and personal level.^[31]^

Scholars have suggested providing training to educational instructors to increase teacher efficacy, decrease teacher stress, reduce teacher truancy, and increase collaborative efforts among teachers.^[32]^ In essence, the continuous reduction in occupational stress among university lecturers in the REBT group from posttest to follow-up indicated a positive effect of REBT. The ability of lecturers in the REBT intervention group to replace irrational beliefs with rational beliefs and use REBT techniques contributed to their positive experiences. Altering irrational beliefs can decrease stress-induced emotional disturbance, as Ellis^[33]^ and other researchers noted. Assisting university lecturers in building rational thoughts and beliefs using REBT coping strategies can help them perform better, maintain good health, cope with stressful situations, and make positive contributions towards societal development.

## 5. Limitations

Like other studies, this study had limitations during the intervention period. Security challenges in the country added stress and burden to researchers and research assistants, prolonging the study’s duration. Additionally, individuals in the waitlist control group did not receive any treatment during the intervention. Despite efforts to ensure smooth intervention, caution should be taken when generalizing the findings. Limiting the scope of this study to a single discipline could impact the conclusions drawn and have implications for the regional scope. Therefore, future studies should explore the geographical scope in subsequent research. Future studies should compare the impact of REBT intervention with other therapeutic techniques used in stress management.

## 6. Strengths

The implementation of REBT for agricultural university lecturers experiencing occupational stress had a positive impact on both their work-related beliefs and stress levels. The questionnaire used to assess teachers’ occupational stress levels in this study has been widely validated. However, challenges related to insecurity in the country could have hindered the potential results of the study. Despite this, the researchers persevered and used these experiences as a starting point.

Additional factors that contributed to the strength of the study included participant randomization, power calculation, hidden allocation, appropriate use of statistical techniques, and the researchers’ ability to control extraneous variables. Ultimately, the study found a favorable impact of REBT on the occupational stress levels of university agricultural lecturers.

## 7. Conclusion

The result of this study validates the usefulness of REBT in decreasing occupational stress among university agricultural lecturers in southeast Nigeria. The outcome of the results shows that lectures exposed to intervention recorded significant in reductions occupational stress in contrast with those who are in the control group. Thus, the findings of this research are imperative in cushioning the cause–effect of occupational stress in our higher institutions of learning especially among the lecturers. In view of this, we recommend that future researchers adopt REBT principles in their professional practices.

## Author contributions

**Conceptualization:** Benedicta Anene Omeje, Ngozi Sandra Ikenye, Felicia Ngozi Ezeaku, Gloria Njideka Ikegbusi, Mary Oluwatoyn Ani.

**Data curation:** Benedicta Anene Omeje, Ngozi Sandra Ikenye, Felicia Ngozi Ezeaku, Emeka Kelechi Ogbonna, Kelechi Ruth Ede, Gloria Njideka Ikegbusi, Mary Oluwatoyn Ani, Bassey Ndubisi Njioku, Njideka Chinwe Ugoji.

**Formal analysis:** Benedicta Anene Omeje, Ngozi Sandra Ikenye, Felicia Ngozi Ezeaku, Emeka Kelechi Ogbonna, Kelechi Ruth Ede, Gloria Njideka Ikegbusi, Mary Oluwatoyn Ani, Bassey Ndubisi Njioku, Njideka Chinwe Ugoji.

**Funding acquisition:** Benedicta Anene Omeje, Ngozi Sandra Ikenye, Felicia Ngozi Ezeaku, Emeka Kelechi Ogbonna, Kelechi Ruth Ede, Ifunanya Nkechi Ohamobi, Gloria Njideka Ikegbusi, Scholastica Uchenna Ekwueme, Godwin E. Eze, Mary Oluwatoyn Ani, Bassey Ndubisi Njioku, Ebele G. Nwoye, Fredrick C. Onah, Lilian U. Ekenta, Njideka Chinwe Ugoji, Samuel Ifeanyi Aba.

**Investigation:** Benedicta Anene Omeje, Ngozi Sandra Ikenye, Felicia Ngozi Ezeaku, Emeka Kelechi Ogbonna, Kelechi Ruth Ede, Ifunanya Nkechi Ohamobi, Gloria Njideka Ikegbusi, Scholastica Uchenna Ekwueme, Mary Oluwatoyn Ani, Bassey Ndubisi Njioku, Ebele G. Nwoye, Lilian U. Ekenta, Samuel Ifeanyi Aba.

**Methodology:** Benedicta Anene Omeje, Ngozi Sandra Ikenye, Felicia Ngozi Ezeaku, Emeka Kelechi Ogbonna, Kelechi Ruth Ede, Ifunanya Nkechi Ohamobi, Gloria Njideka Ikegbusi, Scholastica Uchenna Ekwueme, Mary Oluwatoyn Ani, Bassey Ndubisi Njioku, Ebele G. Nwoye, Lilian U. Ekenta, Samuel Ifeanyi Aba.

**Project administration:** Benedicta Anene Omeje, Ngozi Sandra Ikenye, Felicia Ngozi Ezeaku, Emeka Kelechi Ogbonna, Kelechi Ruth Ede, Ifunanya Nkechi Ohamobi, Gloria Njideka Ikegbusi, Scholastica Uchenna Ekwueme, Bassey Ndubisi Njioku, Ebele G. Nwoye, Fredrick C. Onah, Samuel Ifeanyi Aba.

**Resources:** Benedicta Anene Omeje, Ngozi Sandra Ikenye, Felicia Ngozi Ezeaku, Emeka Kelechi Ogbonna, Kelechi Ruth Ede, Ifunanya Nkechi Ohamobi, Gloria Njideka Ikegbusi, Godwin E. Eze, Bassey Ndubisi Njioku, Lilian U. Ekenta, Njideka Chinwe Ugoji, Samuel Ifeanyi Aba, Robert Nyakuwa.

**Software:** Benedicta Anene Omeje, Ngozi Sandra Ikenye, Felicia Ngozi Ezeaku, Emeka Kelechi Ogbonna, Kelechi Ruth Ede, Ifunanya Nkechi Ohamobi, Gloria Njideka Ikegbusi, Godwin E. Eze, Bassey Ndubisi Njioku, Lilian U. Ekenta, Njideka Chinwe Ugoji, Samuel Ifeanyi Aba, Robert Nyakuwa.

**Supervision:** Benedicta Anene Omeje, Ngozi Sandra Ikenye, Felicia Ngozi Ezeaku, Emeka Kelechi Ogbonna, Kelechi Ruth Ede, Gloria Njideka Ikegbusi, Godwin E. Eze, Mary Oluwatoyn Ani, Ebele G. Nwoye, Fredrick C. Onah, Lilian U. Ekenta, Njideka Chinwe Ugoji, Samuel Ifeanyi Aba, Robert Nyakuwa.

**Validation:** Benedicta Anene Omeje, Ngozi Sandra Ikenye, Felicia Ngozi Ezeaku, Emeka Kelechi Ogbonna, Kelechi Ruth Ede, Ifunanya Nkechi Ohamobi, Gloria Njideka Ikegbusi, Scholastica Uchenna Ekwueme, Godwin E. Eze, Mary Oluwatoyn Ani, Ebele G. Nwoye, Fredrick C. Onah, Lilian U. Ekenta, Njideka Chinwe Ugoji, Samuel Ifeanyi Aba, Robert Nyakuwa.

**Visualization:** Benedicta Anene Omeje, Ngozi Sandra Ikenye, Felicia Ngozi Ezeaku, Emeka Kelechi Ogbonna, Kelechi Ruth Ede, Ifunanya Nkechi Ohamobi, Gloria Njideka Ikegbusi , Scholastica Uchenna Ekwueme, Mary Oluwatoyn Ani, Ebele G. Nwoye, Fredrick C. Onah, Lilian U. Ekenta, Njideka Chinwe Ugoji, Samuel Ifeanyi Aba, Robert Nyakuwa.

**Writing – original draft:** Benedicta Anene Omeje, Ngozi Sandra Ikenye, Felicia Ngozi Ezeaku, Emeka Kelechi Ogbonna, Kelechi Ruth Ede, Ifunanya Nkechi Ohamobi, Gloria Njideka Ikegbusi , Scholastica Uchenna Ekwueme, Godwin E. Eze, Mary Oluwatoyn Ani, Bassey Ndubisi Njioku, Ebele G. Nwoye, Fredrick C. Onah.

**Writing – review & editing:** Benedicta Anene Omeje, Ngozi Sandra Ikenye, Felicia Ngozi Ezeaku, Ifunanya Nkechi Ohamobi, Gloria Njideka Ikegbusi, Scholastica Uchenna Ekwueme, Godwin E. Eze, Mary Oluwatoyn Ani, Bassey Ndubisi Njioku, Ebele G. Nwoye, Fredrick C. Onah, Lilian U. Ekenta, Njideka Chinwe Ugoji, Samuel Ifeanyi Aba, Robert Nyakuwa.
